# A Critical Characteristic in the Transverse Galloping Pattern

**DOI:** 10.1155/2015/631354

**Published:** 2015-02-18

**Authors:** Xiaohui Wei, Yongjun Long, Chunlei Wang, Shigang Wang

**Affiliations:** School of Mechanical Engineering, Shanghai Jiao Tong University, Shanghai 200240, China

## Abstract

Transverse gallop is a common gait used by a large number of quadrupeds. This paper employs the simplified dimensionless quadrupedal model to discuss the underlying mechanism of the transverse galloping pattern. The model is studied at different running speeds and different values of leg stiffness, respectively. If the horizontal running speed reaches up to a critical value at a fixed leg stiffness, or if the leg stiffness reaches up to a critical value at a fixed horizontal speed, a key property would emerge which greatly reduces the overall mechanical forces of the dynamic system in a proper range of initial pitch angular velocities. Besides, for each horizontal speed, there is an optimal stiffness of legs that can reduce both the mechanical loads and the metabolic cost of transport. Furthermore, different body proportions and landing distance lags of a pair of legs are studied in the transverse gallop. We find that quadrupeds with longer length of legs compared with the length of the body are more suitable to employ the transverse galloping pattern, and the landing distance lag of a pair of legs could reduce the cost of transport and the locomotion frequency.

## 1. Introduction

Quadrupeds employ different gaits as speed varies [1–5]. There exist two distinct galloping patterns as the locomotion speed reaches up to a high level, known as the transverse galloping pattern and the rotary galloping pattern. These two different galloping patterns were fully discussed in previous literatures [6, 7], where the transverse gallop is epitomized by horses and the rotary gallop is epitomized by cheetahs. The transverse galloping pattern is employed by many large quadrupeds, for example, the buffalo, horse, camel, and wapiti [2].

Biancardi and Minetti found that slower and larger mammals, with relatively longer and thicker limbs, predominantly employ the transverse gallop, and the aspect ratio (height/body length) was significantly larger than rotary gallopers [8]. The horse that uses the transverse gallop is perhaps the most efficient running machine ever evolved [6, 9]. As the galloping speed increases, the energy consumption per unit distance slightly drops for the horse [3, 9]. Therefore, the transverse galloping pattern might be more suitable for a large, heavy, and long distance running quadruped.

For theoretical analysis, Nanua and Waldron modeled the quadruped as a rigid beam with four massless springy legs [10]. They found that gallop was more efficient than bound, and the range of vertical fluctuation of the center of mass was lesser in a galloping pattern. But they only use one constant leg stiffness and body proportion, and only the rotary gallop was discussed. Herr and McMahon modeled the horse as a two-dimensional numerical model and applied the transverse galloping pattern as the real horse adopts [11]. But only one set of horse parameters was employed in analyzing the dynamic properties. Actually, quadrupeds that utilized the transverse galloping pattern have different sets of body parameters (e.g., the body mass, the body proportion, the leg stiffness, and the leg length), which can affect the overall dynamic properties of the model.

In this study, we employ the simplified dimensionless dynamics of the transverse galloping pattern [10, 12–14] to discuss how the dynamic performance of the transverse gallop can be affected by horizontal running speeds, leg stiffness, body proportions, and landing distance lags of a pair of legs. In order to minimize the interdifferences among different types of quadrupeds, we employ the dimensional analysis [15, 16]. The dimensional analysis is a remarkable tool in so far as it can be applied to any and every quantitative model, no matter how complex the physical model is [13], and it can broaden our analysis of investigating the underlying mechanism of the transverse gallop.

The main aim of this paper is to reveal the intrinsic properties of the transverse gallop using the simplified model, and it can help us to have a better understanding of the transverse galloping pattern that is widely employed by relatively large and heavy quadrupeds. The obtained mechanism information and suggested designing strategies would be a contribution to building the four-legged systems.

## 2. Methods

### 2.1. The Simplified Transverse Galloping Model

**Figure 1:**
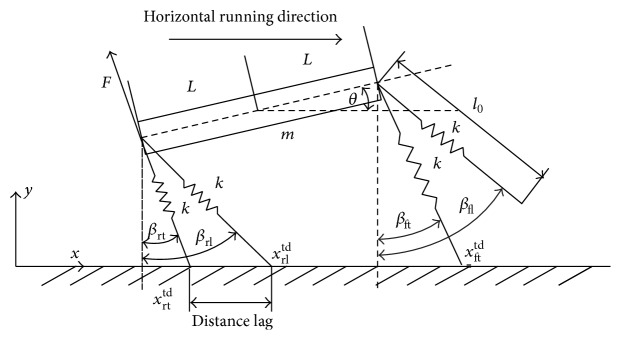
The simplified model for transverse gallop. The topside arrow indicates the direction of running. Each leg is characterized by the stiffness *k*. The uncompressed length of leg is *l*_0_. Two legs are attached to the shoulder joint, and the other two are attached to the hip joint. The leg orientation is characterized by *β*. The body orientation is characterized by *θ*. The length of the half rigid beam is characterized by *L*. *F* represent the leg force and its orientation coincides with the leg orientation.

We employed a simplified transverse galloping model to study its dynamic properties, as shown in Figure 1. The simplified model has been demonstrated to be helpful in capturing important properties of quadruped gaits, such as the trot, the bound, and the gallop [10–14, 17–20]. The simplification of the model can help us to focus on the fundamental mechanism of the galloping pattern. In this model, the body is modeled as a rigid beam [18, 19], where the center of mass is at its geometrical center and the legs were represented as four massless linear springs [12, 13, 21, 22]. The legs of the model can be treated as springs and the inertial effects of the legs are negligible compared to the inertial effects of the body [22]. Two legs are attached to the shoulder joint and the other two are attached to the hip joint. The section of Abbreviations lists the variables and indexes needed to describe the model. The system dynamic equations are obtained by the Lagrangian approach and can be presented as follows:

(1) x¨=−kmcrtl0−lrtsinβrt+crll0−lrlsinβrl   +cftl0−lftsinβft+cfll0−lflsinβfl,y¨=kmcrtl0−lrtcos⁡βrt+crll0−lrlcos⁡βrl   +cftl0−lftcos⁡βft+cfll0−lflcos⁡βfl−g,θ¨=kLJ−crtl0−lrtcos⁡βrt−θ−crll0−lrlcos⁡βrl−θ   +cftl0−lftcos⁡βft−θ   +cfll0−lflcos⁡βfl−θ,

where the body inertia can be calculated by the following equation:

(2) $$\begin{array}{c} J=\frac{1}{12}m{\left(2L\right)}^{2}. \end{array}$$

The length and the angle of each leg when landing on the ground can be obtained by the following equations, where rt, rl, ft, and fl represent the rear trailing leg, the rear leading leg, the front trailing leg, and the front leading leg:

(3)lrtx−Lcos⁡θ−xrttd2+y−Lsinθ2lrl=x−Lcos⁡θ−xrltd2+y−Lsinθ2lft=x+Lcos⁡θ−xfttd2+y−Lsinθ2lfl=x+Lcos⁡θ−xfltd2+y−Lsinθ2βrt=arctanxrttd−x+Lcos⁡θy−Lsinθβrl=arctanxrltd−x+Lcos⁡θy−Lsinθβft=arctanxfttd−x−Lcos⁡θy+Lsinθβfl=arctanxfltd−x−Lcos⁡θy+Lsinθ(4)ci=1the  ith  leg  is  on⁡  the  ground0the  ith  leg  is  not  on⁡  the  ground,i=rt,rl,ft,fl

### 2.2. Dimensionless Dynamic Model

Dimensional analysis can be applied to all quantitative models and offers an efficient way to display complex data sets [13]. Using the dimensional analysis, we can concentrate on the intrinsic properties of the transverse galloping gait without considering the effect caused by different choices of the scales and units of the model. The results would be rather general and indicate the fundamental properties that commonly exist in transverse gallop. Such dimensionless variables are introduced as follows:

(5)t∗tl0/g,(6)x∗=xl0,x˙∗=x˙gl0,x¨∗=x¨g,(7)y∗=yl0,y˙∗=y˙gl0,y¨∗=y¨g,(8)θ∗=θ,θ˙∗=θ˙g/l0,θ¨∗=θ¨g/l0,(9)m∗=mm0,f∗=fg/l0,F∗=Fm0g.

The equation (${\dot{x}}^{*}=\dot{x}/\sqrt{gl_{0}}$) is widely used by experimental biologists. It is known as the Froude number Fr [23], defined as $\text{Fr}=v/\sqrt{gl_{0}}$, where *v* is the forward speed.

The dimensionless inertia *j* [24] and the relative stiffness of the leg (*k* ^*^) [25] are defined as

(10) jJmL2,k∗=kl0m0g.

In order to discuss the influence of the body proportion on performance of the transverse galloping pattern, we introduce the following parameter:

(11) $$\begin{array}{c} p=\frac{2L}{l_{0}}. \end{array}$$

By substituting (6)–(11) into the equations of the dynamics, given by (1)–(3), we can get the dimensionless equation of the system:

(12) x¨∗=−k∗crt1−l∗rtsinβ∗rt+crl1−l∗rlsinβ∗rl   +cft1−l∗ftsinβ∗ft+cfl1−l∗flsinβ∗fl,y¨∗=k∗crt1−l∗rtcos⁡β∗rt+crl1−l∗rlcos⁡β∗rl   +cft1−l∗ftcos⁡β∗ft+cfl1−l∗flcos⁡β∗fl−1,θ¨∗=2k∗pj−crt1−l∗rtcos⁡β∗rt−θ∗       −crl1−l∗rlcos⁡β∗rl−θ∗       +cft1−l∗ftcos⁡β∗ft−θ∗       +cfl1−l∗flcos⁡β∗fl−θ∗,

where

(13) l∗rt=x∗−p2cos⁡θ∗−xrttd∗2+y∗−p2sinθ∗2,l∗rl=x∗−p2cos⁡θ∗−xrltd∗2+y∗−p2sinθ∗2,l∗ft=x∗+p2cos⁡θ∗−xfttd∗2+y∗−p2sinθ∗2,l∗fl=x∗+p2cos⁡θ∗−xfltd∗2+y∗−p2sinθ∗2,β∗rt=arctanxrttd∗−x∗+p/2cos⁡θ∗y∗−p/2sinθ∗,β∗rl=arctanxrltd∗−x∗+p/2cos⁡θ∗y∗−p/2sinθ∗,β∗ft=arctanxfttd∗−x∗−p/2cos⁡θ∗y∗+p/2sinθ∗,β∗fl=arctanxfltd∗−x∗−p/2cos⁡θ∗y∗+p/2sinθ∗.

### 2.3. Transverse Galloping Gait of the Simplified Model

**Figure 2:**
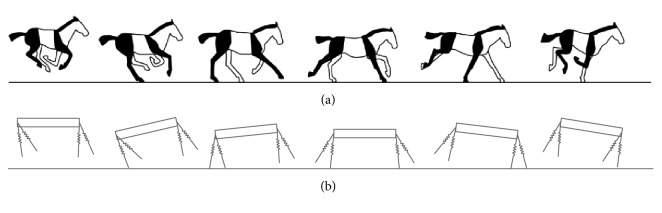
Transverse galloping gait. (a) The equine transverse gallop from the literature [7]. The near side forelimb and hindlimb are indicated in black and the far side is indicated in white. (b) The transverse galloping motion of the model in this paper, which is the same as the real quadrupeds. For we employ the planner dynamic model in this paper, the right and left side of legs are not required for the simulation.

The transverse galloping motion in this paper directly refers to the equine galloping motion [6, 7]. The touchdown sequence of the pair of the front legs is the same as the pair of the rear legs (e.g., touchdown of the right rear leg → touchdown of the left rear leg → touchdown of the right front leg → touchdown of the left front leg, then repeat). There is a typical suspended phase after the front leading leg lifts off the ground, with all of the legs off the ground. As the four legs land on the ground, the front trailing leg remains on the ground before the liftoff of the rear leading leg. We can get detailed description in Figure 2.

### 2.4. Searching Method for the Steady Periodic Gait

The vertical apex point of the center of mass in the fly-phase is set as the initial point in the simulation procedure. After a complete cycle of locomotion, the next apex point appears in the following fly-phase. If the state vector in the first apex point which is represented as **y**_*n*_ is equal to the following state vector (**y**_*n*+1_) in the next apex point, the dynamic system forms a periodical galloping motion [14]. Symmetrical running patterns have been observed in quadrupeds [12, 26, 27]. The model used in this study is symmetric and the searching method could be simplified by using the symmetries.

#### 2.4.1. Symmetries

In this paper, the body symmetries can be expressed as

(14) yfttd∗yrllo∗,yrttd∗=yfllo∗,yfltd∗=yrtlo∗,yrltd∗=yftlo∗,y˙fttd∗=−y˙rllo∗,y˙rttd∗=−y˙fllo∗,y˙fltd∗=−y˙rtlo∗,y˙rltd∗=−y˙ftlo∗,x˙fttd∗=x˙rllo∗,x˙rttd∗=x˙fllo∗,x˙fltd∗=x˙rtlo∗,x˙rltd∗=x˙ftlo∗,θfttd∗=−θrllo∗,θrttd∗=−θfllo∗,θfltd∗=−θrtlo∗,θrltd∗=−θftlo∗,θ˙fttd∗=θ˙rllo∗,θ˙rttd∗=θ˙fllo∗,θ˙fltd∗=θ˙rtlo∗,θ˙rltd∗=θ˙ftlo∗.

The leg symmetries can be expressed as

(15) βfttd∗−βrllo∗,βrttd∗=−βfllo∗,βfltd∗=−βrtlo∗,βrltd∗=−βftlo∗.

#### 2.4.2. Searching Method for Obtaining the Periodic Cycle in the Transverse Galloping Pattern

Transverse gallop has one fly-phase in each locomotion cycle, which starts at the liftoff of the front leading leg and ends at the touchdown of the rear trailing leg. As four legs land on the ground, the two trailing legs remain on the ground before the liftoff of the two leading legs. Hence the searching method needs to find the touchdown angle of the rear trailing leg and touchdown angle of the front trailing leg. The input state vector includes the initial velocity, the initial apex height, the initial pitch angle, the initial pitch angular velocity, the body proportion, and the landing distance lag ratio (Abbreviations).

The dynamic model of this paper is symmetric, and according to the symmetrical requirement, the orientation angle of the rear leading leg is equal to the orientation angle of the front trailing leg at the middle moment of the locomotion cycle:

(16) $$\begin{array}{c} {\beta_{\text{rl}}^{\text{middle}}}^{*}=-{\beta_{\text{ft}}^{\text{middle}}}^{*}. \end{array}$$

Consider the searching variables

(17) $$\begin{array}{c} y^{*}={\left[\begin{array}{cc} {\beta_{\text{rt}}^{\text{td}}}^{*} & {\beta_{\text{ft}}^{\text{td}}}^{*} \end{array}\right]}^{T}. \end{array}$$

Consider the initial input parameters

(18) $$\begin{array}{c} u^{*}={\left[\begin{array}{cccccc} {\dot{x}}^{*} & y^{*} & \theta^{*} & {\dot{\theta }}^{*} & p & \mu \end{array}\right]}^{T}. \end{array}$$

Consider the mapping function

(19) $$\begin{array}{c} \left[\begin{array}{c} {\theta^{\text{middle}}}^{*}\\ {\beta_{\text{rl}}^{\text{middle}}}^{*}+{\beta_{\text{ft}}^{\text{middle}}}^{*} \end{array}\right]=F^{*}\left(y^{*},u^{*}\right). \end{array}$$

The mapping function *F* ^*^ maps the initial input parameters (**u** ^*^) and the initial touchdown angle (**y** ^*^) to

(20) $$\begin{array}{c} \left[\begin{array}{c} {\theta^{\text{middle}}}^{*}\\ {\beta_{\text{rl}}^{\text{middle}}}^{*}+{\beta_{\text{ft}}^{\text{middle}}}^{*} \end{array}\right]. \end{array}$$

If *F* ^*^(**y** ^*^, **u** ^*^) = 0, then the dynamic model forms the periodic gait. To find the initial conditions, we need to solve the equation

(21) $$\begin{array}{c} F^{*}\left(y^{*},u^{*}\right)=0. \end{array}$$

Using the Newton-Raphson method, we can get the iteration equation

(22) $$\begin{array}{c} y_{n+1}=y_{n}+{\left(I-\nabla F\left(y_{n}\right)\right)}^{-1}\left(F\left(y_{n}\right)-y_{n}\right). \end{array}$$

To get the derivative of (22), we adopt the methods used in the previous literature [17]. The central difference approximation using small scalar quantity of 1*e* − 8 is used to perturb each touchdown angle. Equation (22) is evaluated until convergence (max⁡⁡(|*θ* ^middle^^*^|) < 1*e* − 6 and max⁡⁡(|*β*_rl_^middle^^*^ + *β*_ft_^middle^^*^|) < 1*e* − 6).

### 2.5. Initial Parameters and Simulation Tools

The basic stiffness of legs (*k*_0_) is calculated from the formula (*k*_0_ = 0.715*m* ^0.67±0.15^ [28]), which is then multiplied by an adjustment coefficient *k*_*a*_ (ranges from 0.6 to 1.4) as a means to fully discuss the influence of different values of leg stiffness on the dynamic performance of the transverse gallop (*k* = *k*_0_ × *k*_*a*_). The dimensionless leg stiffness is calculated using (10).

The evaluation indicator (1/*L*_c_^*^) represents the metabolic cost of transport (the energy needed to transport a unit weight for a unit distance). It is an estimated prediction for the running simulation [20, 29] and could be applied to predict the cost of transport in dynamic model for different speeds [20]. *L*_c_^*^ is obtained directly from the simulation results, after the searching method obtained the periodic gait. Consider the following.

 The dimensionless inertia is *j* = *J*/*mL* ^2^ = 1/3.
 The body proportion (*p*) ranges from 0.5 to 1.5.
 The lag ratio (*μ*) ranges from 0 to 0.20.

For numerical integration, we used event based ode45 in MATLAB 2011b with absolute and relative error tolerances of 1*e* − 10. The maximum time step was set to 1*e* − 3.

## 3. Results

### 3.1. The Key Property in the Transverse Gallop

**Figure 3:**
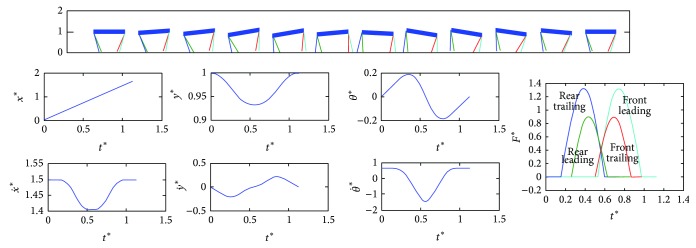
Snapshots and evolutions of states in transverse gallop. Initial conditions: *y* ^*^_s_ = 1, *μ* = 0.05, *p* = 1.0, *k*_*a*_ = 1.0, and *v* ^*^ = 1.5.

**Figure 4:**
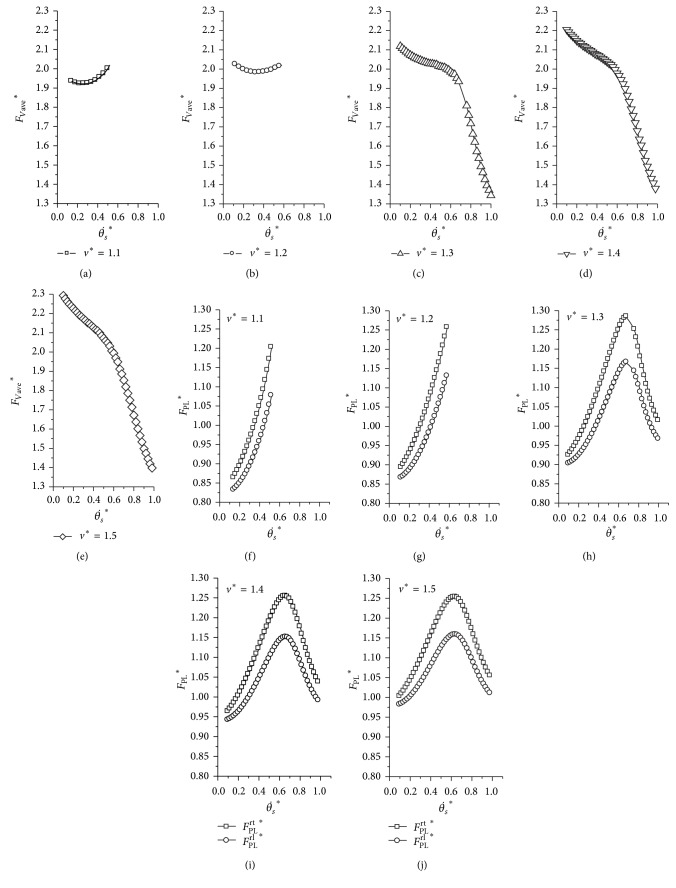
The average vertical force, peak leg force of the rear trailing leg, and the rear leading leg for different dimensionless horizontal speeds. (a)–(e) are the average dimensionless vertical forces for different horizontal velocities, and (f)–(j) are the dimensionless peak leg forces of the rear trailing and the rear leading for different horizontal velocities. Initial conditions: *y* ^*^_s_ = 1, *μ* = 0.05, *p* = 1.0, and *k*_*a*_ = 1.0.

Using the searching methods, we obtained a large number of periodic cycles in the transverse gallop.  Figure 3 presents evolutions of the states during one locomotion cycle. The galloping motion exhibits symmetric properties, which is consistent with previous results [26]. Then, we simulated the transverse gallop model under different dimensionless horizontal velocities. As the horizontal speed increases, the dynamics presents two different situations.  Figure 4 is one of the obtained periodic cycles to describe this feature.

When the dimensionless horizontal speed is relatively low, the transverse galloping model can only obtain such initial parameters that lead to small changes of the average vertical force (*F*_Vave_^*^) and the peak leg force (*F*_PL_^*^) with the varying initial pitch angular velocities (${{\dot{\theta }}_{s}^{\;}}^{*}$). As the transverse galloping motion is symmetric, the peak leg force of the rear trailing leg is equal to the peak leg force of the front leading legs, as the other two legs (the rear leading leg and the front trailing leg). Therefore, only the peak leg forces of the rear legs are considered in this paper. With an increase of the horizontal speed (*v* ^*^ ≥ 1.3 in Figure 4), there emerges a particular situation in which the average vertical force and peak leg force sharply decline as the initial pitch angular velocity (${{\dot{\theta }}_{s}^{\;}}^{*}$) increases. For example, when the dimensionless horizontal speed is 1.1, the *F*_Vave_^*^ is at a high level, and the *F*_PL_^*^ of the two rear legs increases with the ${{\dot{\theta }}_{s}^{\;}}^{*}$ (Figures 4(a) and 4(f)). However, when the horizontal speed reaches up to 1.3, the *F*_Vave_^*^ sharply decreases with an increase of ${{\dot{\theta }}_{s}^{\;}}^{*}$, as the *F*_PL_^*^ (Figures 4(c) and 4(h)).

### 3.2. Influence of Different Values of Leg Stiffness on the Transverse Gallop

We simulate the transverse gallop under different *k*_*a*_ at the same dimensionless horizontal speed to investigate how different values of leg stiffness would affect the dynamic performance of the transverse galloping pattern.

**Figure 5:**
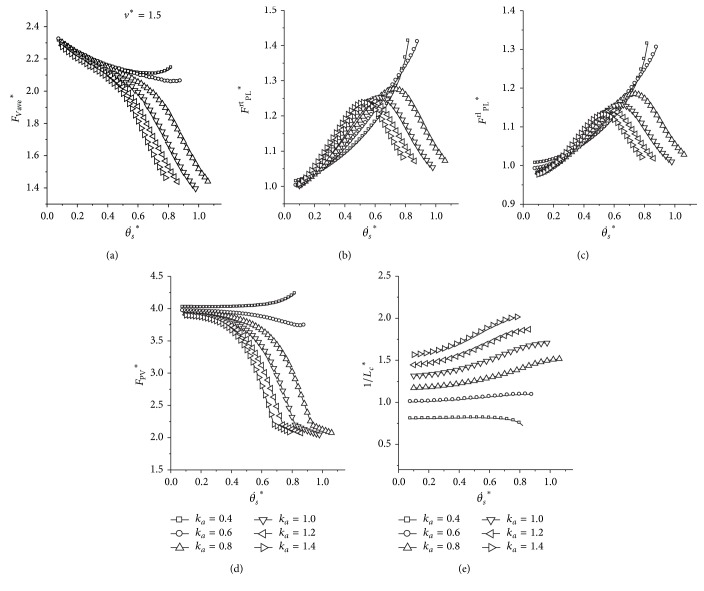
The influence of different *k*_*a*_ on the dynamics. (a) Average dimensionless vertical force; (b) peak force of the rear trailing leg; (c) peak force of the rear leading leg; (d) peak ground reaction force; (e) indicator of the metabolic cost of transport (1/*L*_c_^*^). Initial conditions: *y* ^*^_s_ = 1, *μ* = 0.05, *p* = 1.0, and *v* ^*^ = 1.5.

It is indicated from Figure 5 that the average vertical force (*F*_Vave_^*^) decreases slightly with an increase of the initial angular velocity when the adjustment coefficient of the leg stiffness (*k*_*a*_) is small (*k*_*a*_ = 0.4, 0.6). However, when the leg stiffness reaches up to a critical value (*k*_*a*_ ≥ 0.8), as shown in Figure 5, a completely distinct performance emerges with an increase of the initial angular velocity (${{\dot{\theta }}_{s}^{\;}}^{*}$). The *F*_Vave_^*^ and *F*_PV_^*^ sharply reduce to a relatively low level with the increasing ${{\dot{\theta }}_{s}^{\;}}^{*}$. The peak leg forces increase firstly and then decrease as the initial pitch angular velocity increases. Generally, the metabolic cost of transport increases with an increase of the *k*_*a*_, as shown in Figure 5(e).

### 3.3. The Scope of the Key Property

A large number of periodic cycles are found by using the above methods. This key property is prevalent in transverse galloping model if the running speed reaches up to a critical value (*k*_*a*0_). The transverse galloping model would possess such key property if *k*_*a*_ is equal to or greater than *k*_*a*0_; nevertheless, if *k*_*a*_ is less than *k*_*a*0_, the dynamics system will not appear to have such property.

**Figure 6:**
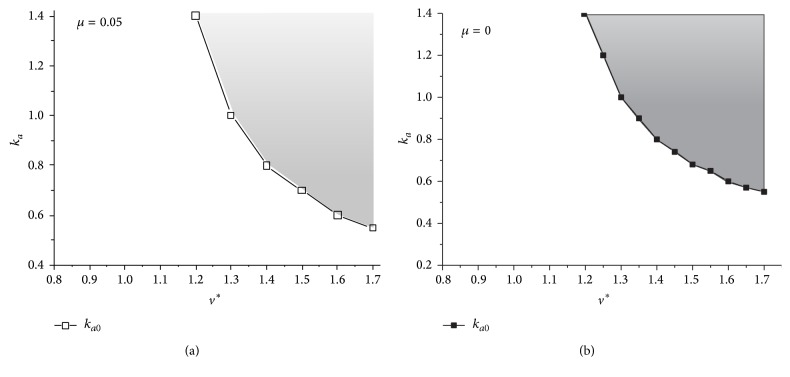
The scope of *k*_*a*_ for the emergence of the key property. The grey area represents the set of parameters (*k*_*a*_, *v* ^*^) that enable the model to possess the key property. Initial conditions: *y* ^*^_s_ = 1 and *p* = 1.0.

With an increase of the dimensionless horizontal velocity, the corresponding *k*_*a*0_ decreases, as shown in Figure 6(a). When the horizontal speed reaches up to a critical value at a fixed *k*_*a*_, such key property that greatly reduces the loads the model endures would always be maintained. For example, for a fixed leg stiffness (*k*_*a*_ = 1.0), when the horizontal speed is less than 1.3, the transverse galloping model does not possess this key property. As the horizontal speed increases to 1.3 or more than 1.3, the dynamic system would always possess this key property. This critical value (*k*_*a*0_) separates the dynamics performance of the transverse gallop into two distinct situations. If the lag ratio (*μ*) is equal to zero, the rear legs and front legs would maintain the same phase and rhythm. In other words, the rear pair of legs and the front pair legs would touch and lift off the ground at the same time, which would generate a bounding gait. As shown in Figure 6(b), this key property also exists in the bounding gait.

### 3.4. The Optimal Option of *k*_*a*_ with the Increasing Horizontal Speed

From the results in Sections 3.1, 3.2, and 3.3, the key property would emerge if the running speed reaches up to a critical value at a fixed leg stiffness, and the key property emerges as the leg stiffness reaches a critical value at a fixed horizontal velocity. We consider both the mechanical loads that the dynamic system endures and the metabolic cost of transport to see if there is an optimal set of parameters that can not only let the dynamic system endure the minimum mechanical force, but also consume the minimum energy.

From the results in Section 3.2, when the adjustment coefficient of the leg stiffness (*k*_*a*_) reaches up to 0.8, the dynamic system possesses the key property, and with an increase of *k*_*a*_, the metabolic cost of transport decreases. Moreover, the minimum mechanical forces are nearly the same. In other words, the parameters at the boundary that separates the white and grey area in Figure 6 would enable the dynamic system to have the optimal gait considering both the mechanical loads and the metabolic cost of transport.

**Figure 7:**
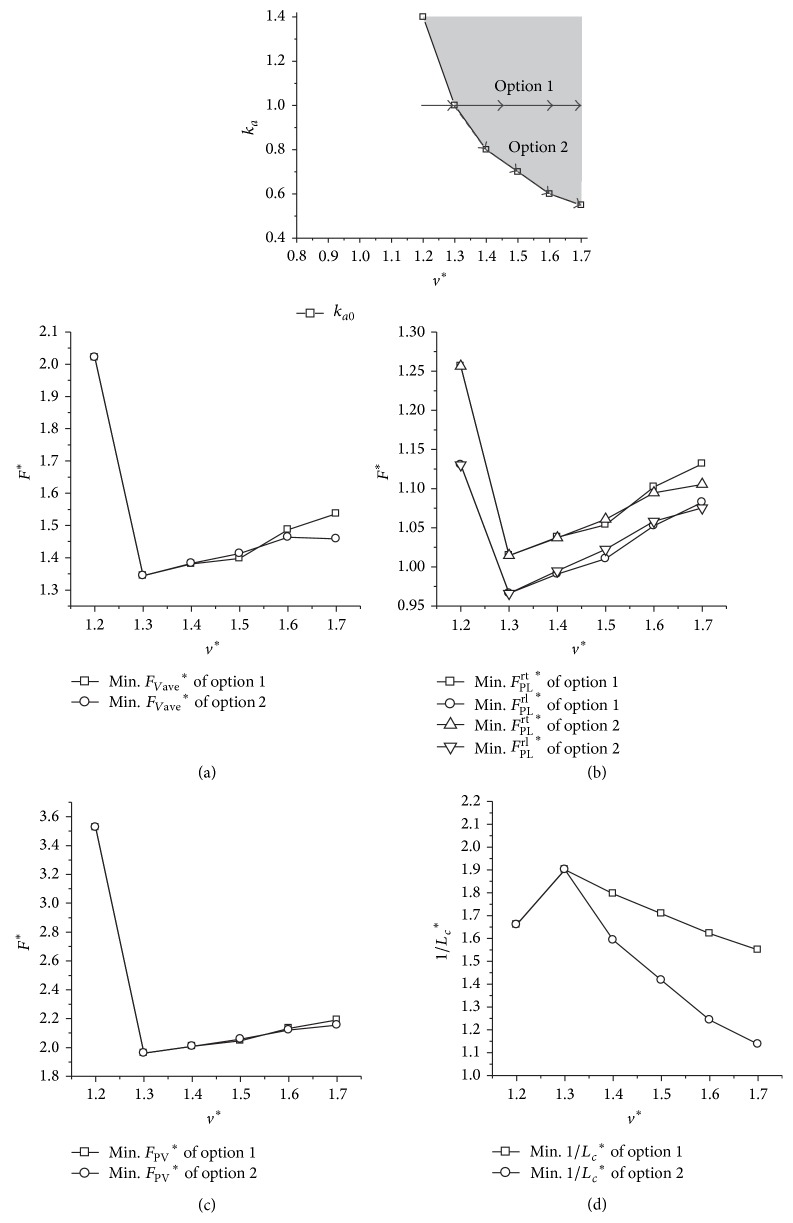
The performances of the dynamic system under two different options of *k*_*a*_. (a), (b), and (c) represent the results of the average vertical force, peak leg force, and peak ground reaction force, respectively. (d) is the result of the indicator of the metabolic cost of transport. Initial conditions: *y* ^*^_s_ = 1, *μ* = 0.05, and *p* = 1.0.

Two ways of options of *k*_*a*_ are designed to present the difference (Figure 7). The leg stiffness remains the same regardless of the varying speeds in option 1, while, in option 2, the leg stiffness varies with the horizontal speeds and the *k*_*a*_ lies in the boundary. It is shown in Figure 7 that mechanical loads in the white area are much higher than those in the grey area (Figures 7(a), 7(b), and 7(c)). The mechanical loads of option 1 and option 2 are almost the same (Figures 7(a), 7(b), and 7(c)) in the grey area where the key property emerges; however, the metabolic cost of transport is smaller in option 2 than in option 1. This difference is getting more and more obvious with an increase of the horizontal speed (Figure 7(d)).

### 3.5. Influence of the Body Proportion (*p*) on the Dynamic Performance

For the sake of concentrating on how the performance of the transverse gallop would be affected by the body proportion, we tested three values of *p*, and they are all simulated in the sets of initial parameters where the key property emerges.

**Figure 8:**
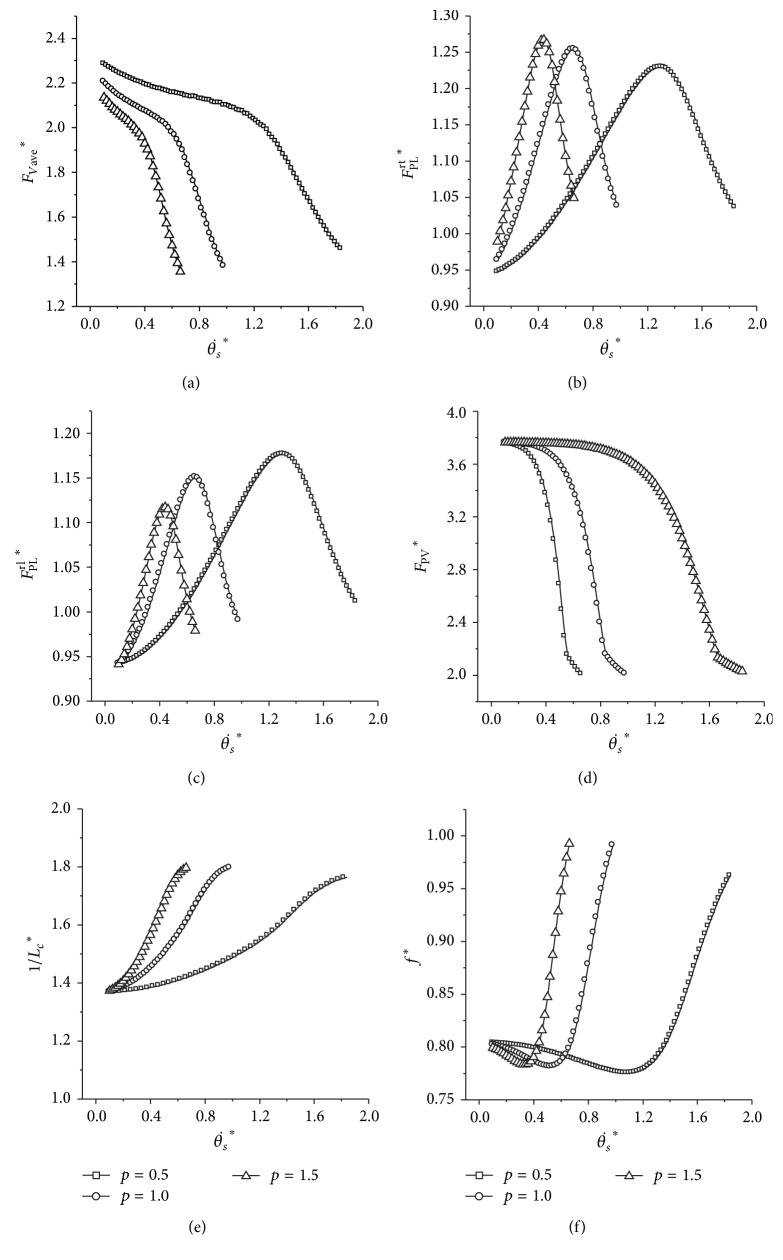
Influence of different body proportions on the transverse galloping model. (a) The average vertical force; (b) and (c) are the peak leg force for the rear trailing and the leading legs, respectively. (d) The peak ground reaction force; (e) the result of the indicator of the metabolic cost of transport; (f) the locomotion frequency. Initial conditions: *y* ^*^_s_ = 1, *μ* = 0.05, *v* ^*^ = 1.4, and *k*_*a*_ = 1.0.

From Figure 8, with a decrease of the body proportion (*p*), the minimum *F*_Vave_^*^ and the minimum *F* ^rl^_PL_^*^ increase slightly (Figures 8(a) and 8(c)), and the minimum *F* ^rt^_PL_^*^ decreases slightly (Figure 8(b)). The minimum peak ground reaction force (*F*_PV_^*^) remains nearly the same regardless of the varying body proportions (Figure 8(d)). The metabolic cost of transport and the frequency of locomotion would maintain a low level for a smaller body proportion (*p*). For example, when the dimensionless initial angular velocity is 0.66, the indicators of the metabolic cost of transport (1/*L*_c_^*^) are 1.79696, 1.60457, and 1.41816 for *p* = 1.5, 1.0, and 0.5, respectively (Figure 8(e)).

**Figure 9:**
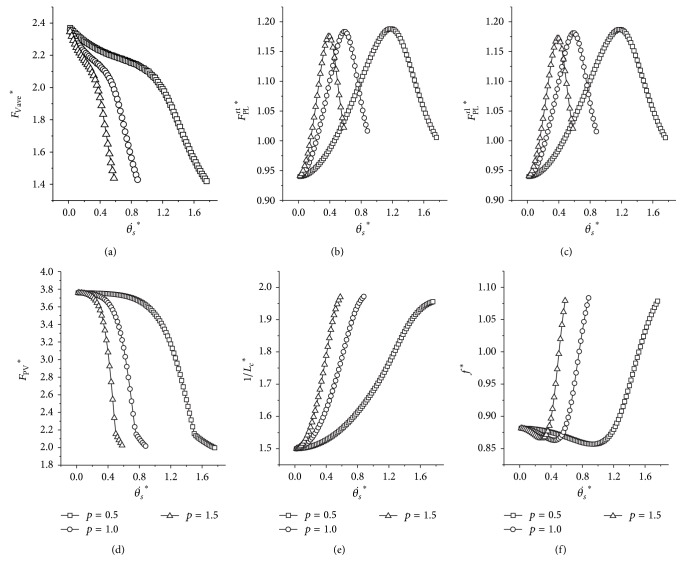
Influence of different body proportions on the bounding model. (a) The average vertical force; (b) and (c) are the peak leg force for the rear trailing and the leading legs, respectively; (d) the peak ground reaction force; (e) the result of the indicator of the metabolic cost of transport; (f) the locomotion frequency. Initial conditions: *y* ^*^_s_ = 1, *μ* = 0, *v* ^*^ = 1.4, and *k*_*a*_ = 1.0.

**Figure 10:**
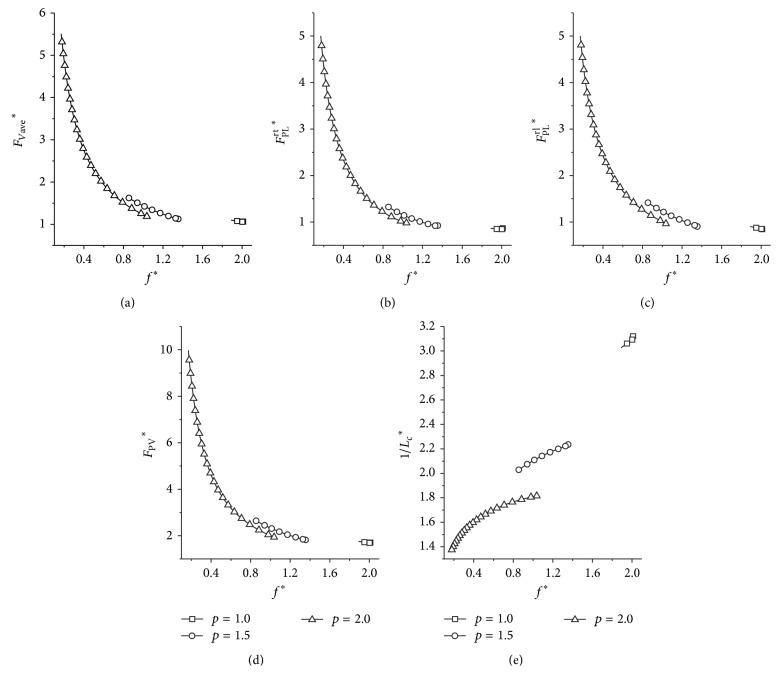
Influence of different body proportions on the rotary galloping model. (a) The average vertical force; (b) and (c) are the peak leg force for the rear trailing leg and the leading leg, respectively. (d) The peak ground reaction force; (e) the result of the indicator of the metabolic cost of transport. Initial conditions: *y* ^*^_s_ = 1, *μ* = 0.05, *v* ^*^ = 1.4, and *k*_*a*_ = 1.0.

The situation when the landing distance lag is equal to zero is also simulated to test if the bounding gait also holds this feature as the transverse gallop does. It is shown in Figure 9 that the bounding gait possesses the similar properties compared with the transverse galloping gait. In a word, the smaller body proportion (*p*) benefits a galloping gait with one fly-phase. For a galloping gait with two fly-phases (rotary galloping gait, the simulation method refers to the previous paper [17]), the minimum values of the average vertical force, the peak leg force, and the peak ground reaction are almost the same (Figures 10(a), 10(b), 10(c), and 10(d)), while the model with the biggest body proportion (*p*) has the minimum corresponding locomotion frequency. Moreover, the metabolic cost of transport is lower for a model with bigger body proportion. Hence, a bigger body proportion benefits a galloping gait with two fly-phases.

### 3.6. Effect of the Lag Ratio (*μ*) on the Dynamic Performance of the Transverse Gallop

There exist landing distance lags in the rear and the front pair of legs in galloping gaits. Why this landing distance lag is generally employed by quadrupeds has not been directly discussed. In this paper, we let the transverse galloping model employ different lag ratios (*μ*) to investigate how the dynamic performance would be affected by this factor.

**Figure 11:**
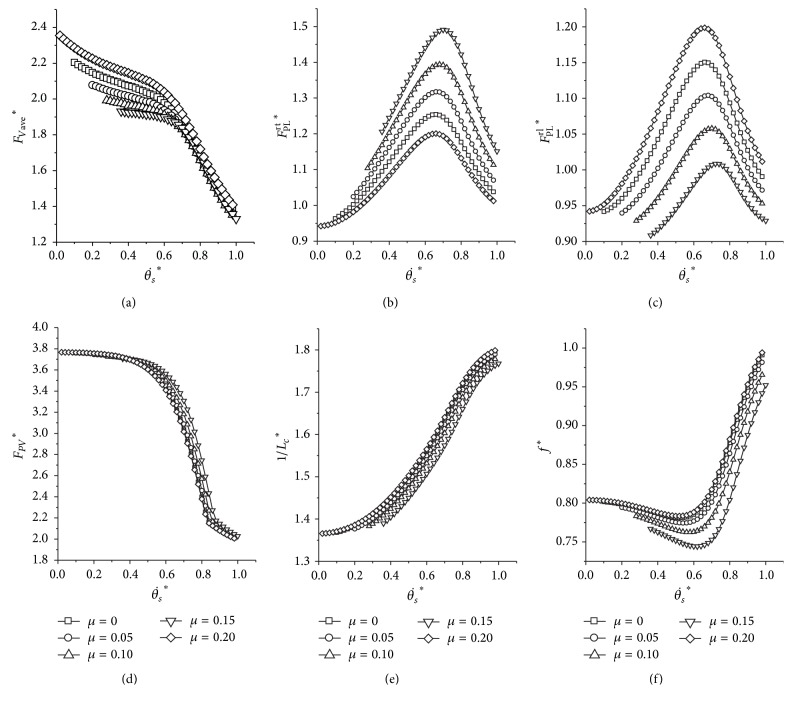
Influence of different lag ratios on the transverse galloping dynamics. (a) The average vertical force; (b) and (c) are the peak leg force for the rear trailing leg and the rear leading leg, respectively. (d) The peak ground reaction force; (e) the result of the indicator of the metabolic cost of transport; (f) the locomotion frequency. Initial conditions: *y* ^*^_s_ = 1, *p* = 1.0, *v* ^*^ = 1.4, and *k*_*a*_ = 1.0.

The minimum *F*_Vave_^*^ and minimum *F*_PV_^*^ maintain almost the same value with the varying lag ratio (Figures 11(a) and 11(d)). The *F* ^rt^_PL_^*^ increases as the lag ratio becomes larger; in the meantime the *F* ^rl^_PL_^*^ decreases (Figures 11(b) and 11(c)). For the peak leg force of the front pair, the peak leg force of the front leading leg is equal to the peak leg force of the rear trailing leg (*F* ^fl^_PL_^*^ = *F* ^rt^_PL_^*^), as the other two legs (*F* ^ft^_PL_^*^ = *F* ^rl^_PL_^*^). As for the metabolic cost of transport and the locomotion frequency, it is indicated from Figures 11(e) and 11(f) that they all decline with an increase of the lag ratio at the same initial angular velocity.

## 4. Discussion

### 4.1. What Leads to the Landing Distance Lag in the Transverse Gallop?

In the transverse galloping pattern, why do quadrupeds utilize landing distance lag in the rear and the front pairs of legs? In Section 3.6, it is shown that if the transverse galloping model adopts a distance lag, the average vertical force and the peak ground reaction force remain nearly the same, but the metabolic energy of transport would decrease with an increase of the lag ratio at the same initial pitch angular velocity. Besides, the locomotion frequency would be much lower. This property is important for the large and heavy quadrupeds that employ the transverse galloping pattern as their fast running gait. Using a larger lag ratio, the energy consumption for moving a unit distance will be much smaller; also the minimum mechanical loads that the mammals endure remain the same. Moreover, when the lag ratio is equal to zero (*μ* = 0), it is another commonly used gait known as the bounding gait; the rear pair and the front pair land on the ground at the same time. The energy consumption of transport of the bounding gait is more than that of galloping at the same initial pitch angular velocity; this is a reason why the transverse galloping pattern would be more favourable than bounding at high running speed.

### 4.2. What Leads to Quadrupeds Changing Their Leading Leg during the Transverse Gallop?

Distance lag during transverse gallop could enable the dynamic system to have lower energy consumption, but the side effect is that the peak leg forces of the rear trailing leg and the front leading leg become higher, and the peak leg forces of the other two legs become lower. The more the lag ratio is, the larger the difference of force between the trailing and leading legs of one pair would become. If quadrupeds gallop at a high speed without varying the leading leg which bears the most loads, the leading leg might be damaged by a relatively long time running. Biewener et al. found that magnitude of stress is greater in the leading leg [30]. Horses that utilize the transverse gallop would mostly fracture their leading leg of the front pair [31]. Biancardi and Minetti found that 48 ± 20 strides were covered between two successive lead leg changes on straights [32]. Bioexperiments showed that horses would change their leading leg during straight running, and the most probable fractured leg is the leading leg of the front pair. This is consistent with the results in this paper. Therefore, quadrupeds might change their leading leg during the transverse gallop to balance the loads endured by legs of the same pair.

### 4.3. Why Is Body Proportion Lower in a Transverse Galloper?

Biancardi and Minetti found that the aspect ratio (height/body length) was significantly lower in rotary than in transverse gallopers [8]. In other words, transverse gallopers own a much higher ratio (height/body length). Why this happens to a transverse galloper is not discussed in that paper. In our works, the body proportion (*p*) is the inverse of the aspect ratio (height/body length). From the result in Section 3.5, the peak forces of the rear trailing leg (*F* ^rt^_PL_^*^) and the rear leading leg (*F* ^rl^_PL_^*^) are lower for a smaller body proportion (*p*) at relatively lower initial angular velocities. At the same time, the metabolic cost of transport is much lesser for a smaller body proportion (*p*). The dynamic system with the smallest *p* possesses the largest range of initial angular velocity that keeps the system in a low value of locomotion frequency. Accordingly, a transverse galloper with a lower body proportion would have a lower peak leg force and energy consumption of transport. For the rotary galloping pattern, the minimum mechanical loads of different body proportions are virtually the same, while the model with the larger body proportion possesses the minimum metabolic energy consumption. Hence, a rotary galloper would be more efficient with a larger proportion. The findings are consistent with the experimental results [8]. It is extremely favourable for large, heavy, and long distance running quadrupeds to run with a lower body proportion, which would reduce the mechanical loads and the energy consumption.

### 4.4. Leg Stiffness Varies with the Horizontal Speed

As for real quadrupeds, the leg stiffness does not alter a lot during locomotion, nearly independent with the running speed [21, 28, 33]. Results in Section 3.5 indicate that if the adjustment coefficient of the leg stiffness (*k*_*a*_) lies in the boundary and varies with the forward horizontal speed, the metabolic cost of transport would be lower. We could not change the musculoskeletal system of the real quadrupeds, but this property could be used in designing the four-legged robotic control system. Robotic system could utilize a little control effort to alter the leg stiffness with the running speed, which does not affect the mechanical characteristic, and obtain the minimum cost of transport. This is a good choice for the high-speed, long distance, and heavy-loaded four-legged systems. In addition, the key property could greatly reduce the mechanical forces and the strength requirement for designing the leg, provided the four-legged system gallops using the parameters of the grey area (Figure 5).

## 5. Conclusions

The present study is helpful for getting a better understanding of the underling mechanics of the transverse galloping pattern that is widely employed by quadrupeds. When the horizontal speed and the leg stiffness reach up to critical values, the transverse galloper could make full use of their dynamics to greatly reduce the mechanical loads that exert the musculoskeletal system. Quadrupeds with a much lower body proportion would be fitter for the transverse gallop, and the landing distance lag of a pair of legs is a method to reduce the metabolic cost of transport. For a four-legged running system designing, it is suggested that the leg stiffness should change with the running speed to reduce the metabolic cost of transport while maintaining much lower mechanical loads.

## Supplementary Material

In order to better illustrate the periodic gait of the transverse galloping pattern obtained using the searching method (Section 2.4), we attached a video showing the motion of the transverse galloping. The initial conditions: *y* ^*^_s_ = 1, *μ* = 0.05, *p* = 1.0, *k*_*a*_ = 1.0, ν^*^ = 1.5.

## Abbreviations

### Variables and Indexes Used in This Study

*x*, *y*: Rectangular coordinates of the center of mass (m)
*k*: The stiffness of legs, *k* = *k*_0_ · *k*_*a*_ (N m^−1^)
*v*: Horizontal running velocity (m/s)
*k*_0_: Basic stiffness of the leg (N m^−1^)
*k*_*a*_: The adjustment coefficient of the stiffness of legs
*k*_*a*0_: The minimum adjustment coefficient enabling the model to possess the key property
*l*: The length of legs (m)
*l*_0_: The free length of legs (m)
*m*: Body mass (kg)
*L*: The half length of the body (m)
*p*: The body proportion, which is the ratio of the length of the body to leg free length (2*L*/*l*_0_)
*θ*: Body orientation angle (rad)
*J*: Body inertia (kg m^2^)
*j*: Dimensionless body inertia
*β*: Leg orientation angle (rad)
*x*^td^: The touchdown position (m)
*g*: Acceleration of gravity (m s^−2^)
*F*_PV_: Peak ground reaction force (N)
*F*_PL_: Peak leg force (N)
*F*_Vave_: Average vertical force from the ground, which is calculated by *I*_ver_/*t*_*c*_ (N)
*I*_ver_: Total vertical impulse from the ground (N s)
*t*_*c*_: The average time of a leg landing on the ground during a cycle (s)
*L*_*c*_: The average distance moved when a leg lands on the ground during a cycle (m)
*μ*: The ratio of the landing distance lag of a pair of legs (rear or front) to the length of the body
*r*: As index: rear leg
*f*: As index: front leg
td: As index: the instant of touchdown
lo: As index: the instant of liftoff
*t*: As index: trailing leg
*l*: As index: leading leg
∗: As superscript: dimensionless.
